# Nesting doctoral students in collaborative North–South partnerships for health systems research

**DOI:** 10.3402/gha.v7.24070

**Published:** 2014-07-15

**Authors:** Svetla Loukanova, Helen Prytherch, Antje Blank, Els Duysburgh, Göran Tomson, Lars L. Gustafsson, Ali Sié, John Williams, Melkizedeck Leshabari, Walter E. Haefeli, Rainer Sauerborn, Sharon Fonn

**Affiliations:** 1 Department of Public Health, University of Heidelberg, Heidelberg, Germany; 2 Department of Clinical Pharmacology and Pharmacoepidemiology, Internal Medicine Department, Heidelberg University Hospital, Heidelberg, Germany; 3 International Centre for Reproductive Health (ICRH), Ghent University, Ghent, Belgium; 4 Department of Learning, Informatics, Management and Ethics, Medical Management Centre (MMC), Karolinska Institutet, Stockholm, Sweden; 5 Department of Public Health Sciences, Health Systems and Policy, Karolinska Institutet, Stockholm, Sweden; 6 Department of Laboratory Medicine, Division of Clinical Pharmacology, Karolinska Institutet at Karolinska University Hospital Huddinge, Stockholm, Sweden; 7 Centre de Recherche en Santé de Nouna, Nouna, Burkina Faso; 8 Navrongo Health Research Centre, Navrongo, Ghana; 9 Department of Behavioral Sciences, School of Public Health and Social Sciences, Muhimbili University of Health and Allied Sciences, Dar es Salaam, Tanzania; 10 Faculty of Health Sciences, School of Public Health, University of the Witwatersrand, Johannesburg, South Africa

**Keywords:** collaborative project, doctoral students, health systems research capacity, North–South Partnership

## Abstract

**Background:**

The European Union (EU) supports North–South Partnerships and collaborative research projects through its Framework Programmes and Horizon 2020. There is limited research on how such projects can be harnessed to provide a structured platform for doctoral level studies as a way of strengthening health system research capacity in sub-Saharan Africa (SSA).

**Objective:**

The aim of this study was to explore the challenges of, and facilitating factors for, ‘nesting’ doctoral students in North–South collaborative research projects. The term nesting refers to the embedding of the processes of recruiting, supervising, and coordinating doctoral students in the overall research plan and processes.

**Design:**

This cross-sectional qualitative study was undertaken by the EU-funded QUALMAT Project. A questionnaire was implemented with doctoral students, supervisors, and country principal investigators (PIs), and content analysis was undertaken.

**Results:**

Completed questionnaires were received from nine doctoral students, six supervisors, and three country PIs (86% responses rate). The doctoral students from SSA described high expectations about the input they would receive (administrative support, equipment, training, supervision). This contrasted with the expectations of the supervisors for proactivity and self-management on the part of the students. The rationale for candidate selection, and understandings of the purpose of the doctoral students in the project were areas of considerable divergence. There were some challenges associated with the use of the country PIs as co-supervisors. Doctoral student progress was at times impeded by delays in the release of funding instalments from the EU. The paper provides a checklist of essential requirements and a set of recommendations for effective nesting of doctoral students in joint North–South projects.

**Conclusion:**

There are considerable challenges to the effective nesting of doctoral students within major collaborative research projects. However, ways can be found to overcome them. The nesting process ultimately helped the institutions involved in this example to take better advantage of the opportunities that collaborative projects offer to foster North–South partnerships as a contribution to the strengthening of local research capacity.

 The countries in sub-Saharan Africa (SSA) continue to face significant health challenges caused by malaria, child malnutrition, maternal and neonatal diseases, persisting infections such as tuberculosis and HIV/AIDS, and the growing burden of non-communicable diseases (1). Despite progress, the overall advance towards achieving the Millennium Development Goals (MDGs) is not on track. Low capacity and lack of resources are the root causes of the functional and organisational failings of health systems that impede progress (1–4). Policy makers in countries with weak public health research capacity struggle to effectively analyse, plan for, and ultimately strengthen health systems (5–7). Moreover, they face challenges to achieve continuous and systematic capacity building for medical and nursing education, master's programmes, research capacity development, management, and leadership education (8–10).

There is peer-reviewed and grey literature available showing different approaches to strengthening research capacity in SSA in cooperation with universities in the Northern hemisphere often in Europe or the United States (so called North–South Partnership). Such approaches include networking among global health researchers (11), institutional collaboration on health systems research capacity development (12), initiatives for specific diseases and programmes, such as mental illness (13), or other critical global health problems (ICER-International Centres for Excellence in Research). More specifically, there are different existing doctoral training models for research capacity building, including those which are African led, (e.g. The African Economics Research Consortium), or supported by research institutions such as the German Research Foundation, DFG (14). Other programmes are facilitated by bi-lateral or multilateral agencies often through a combination of facilitating partnerships between a research institution in SSA with a university in the North, as well as the provision of scholarships for students to undertake their PhD in the North. These approaches all provide a structured research and training environment for doctoral students.

Through its Framework Programmes 6 and 7 as well as Horizon 2020, the European Union (EU) funds collaborative research projects between European academic institutions and research centres and universities in low- and middle- income countries. The role of these programmes as a way to strengthen research capacity is already recognised by the participating institutions and the scientific community (4, 12). However, there is little literature on how collaborative implementation research projects provide a structured platform to accommodate, supervise and coordinate doctoral students as a contribution to building health system research capacity in SSA. The present study explores this poorly investigated issue of how doctoral students can be nested in implementation research collaborations between European universities and research institutions in SSA. Nesting refers to the recruitment of PhD students and placing them, with their own discreet research question, within already funded research collaboration. The PhD students use part of the data for their PhD and are supervised by members within or connected to (at the same university) the research collaboration institutions.

This paper presents the experiences of a group of 10 doctoral students in a major 5-year collaborative project (2009–2014), funded by the EU (Quality of prenatal and maternal care: Bridging the know–do gap – QUALMAT), which seeks to improve the quality of prenatal and maternal care in selected primary health care facilities in rural Burkina Faso, Ghana, and Tanzania. QUALMAT is piloting a computer-assisted clinical decision support system (CDSS) designed to improve clinical decision-making and a system of performance-based incentives intended to enhance health worker motivation (15, 16). This study was conceived one and a half years before the official end of the project at the suggestion of the project's Scientific Advisory Board (see below), which was impressed by the high number of doctoral students and thought that documenting the experience would help the project implementers make some improvements that would also be of relevance to a wider audience.

To address the QUALMAT research agenda, teams of researchers from across the different institutions in the QUALMAT consortium were arranged into Work Packages. Each Work Package had different focus areas and study questions. From the outset there was a strong interest across the consortium to encourage young scientists – especially those from the SSA institutions – to become driving forces within their teams thus the doctoral students were seen as an important component of the Work Package teams. Moreover, it was intended that the doctoral student's attention would be focused on this project, unlike the more experienced collaborators from SSA who are usually involved in several such endeavours concurrently precisely due to the shortage of experienced staff at such institutions. It was also understood that investing in doctoral students is a relatively cost-effective way to use funding. Doctoral students have been found to readily accept modest stipends if in addition their field work, conference participation and publishing costs are covered. While the requirements of each university hosting a PhD student were considered, the influence these would have on coordinating the doctoral students as a group within the project frame was underestimated. Indeed, the substantial variation that exists between European universities regarding the requirements made on doctoral students has not been previously discussed in the context of North–South research collaboration.

At the project outset, a Steering Committee was established to oversee implementation of the research activities, including the doctoral students. Furthermore, international technical experts were invited to serve on a Scientific Advisory Board to guide the project collaborators on scientific and methodolgical issues. Finally, a Policy Advisory Board was set up in each country comprising experts from ministries of health and local government to ensure the interventions were compatible with the respective contexts and prevailing policies, and to help bridge the research-to-policy and policy-to-action gaps (16).

The overall objective of the study presented here was to explore the challenges to and facilitating factors for nesting doctoral students in collaborative projects between universities and research centres in Europe and SSA, and the potential these projects have for improving research capacity. Specific objectives included: 1) to describe the expectations of students and supervisors regarding the doctoral studies process and 2) to identify challenges and facilitating factors surrounding the nesting of doctoral level research in on-going EU-funded research implementation projects.

As described, the study was undertaken to elucidate if this kind of approach to doctoral training was beneficial and how it could be strengthened in the future. It was anticipated that the findings of the study would be of interest and relevance to other institutions engaged in the training of doctoral students nested in collaborative health systems research projects.

## Methods

The Country PIs in the three sub-Saharan African countries identified the doctoral candidates at the start of the project. In general, there was a preference to register the doctoral students at one of the European collaborating partner universities. The collaborating research centres in Burkina Faso and Ghana are not universities and cannot confer degrees. One student from Ghana developed a research idea during the project and registered at the Kwame Nkrumah University of Science and Technology in Ghana.

The doctoral students were assigned to the particular Work Packages according to their backgrounds and the research areas being explored: two students were nested in the Work Package that examined the quality of care and services before and after the introduction of the QUALMAT interventions; two were involved in carrying out an economic evaluation of the interventions; two were involved in research comparing health workers motivation before and after the project interventions; one student did research on the design and implementation of performance-based incentive schemes; and three students were involved in aspects of research related to the design, development and implementation of the computer-based CDSS. Table 1 presents the distribution of the doctoral students across the different Work Packages with their professional background, their host university, and the status of their studies at the time of writing. It shows how the 10 doctoral students were enrolled at different institutions of the consortium. All students except one were from SSA. The average age of the students was 34.7 years (range 27–36 years). The ratio of females to males was 4:6. Six of the students were enrolled at the University of Heidelberg, Germany; two at Ghent University, Belgium; one at the Karolinska Institute, Stockholm, Sweden; as mentioned, one at the Kwame Nkrumah University of Science and Technology, Kumasi, Ghana. This student only received financial support from the project.

**Table 1:** Demographic characteristics of those involved in the project: doctoral students, working on topics and registration at a partner university

No	Gender/age	Institution and country of origin	Professional background	Doctoral research topic	Main university of enrolment	Status of the doctoral work at the time of survey
1	Female	Navrongo Health Research Centre, Navrongo, Ghana	Nurse	The impact of a clinical decision support system and a performance-based incentive approach on the quality of basic emergency obstetric and new-born care provided in selected rural health facilities in Northern Ghana.	International Centre for Reproductive Health, Ghent University, Ghent, Belgium	Finalisation of the research protocol
2	Male	Muhimbili University of Health and Allied Sciences, School of Public Health and Social Sciences, Dar Es Salaam, Tanzania	Medicine	Underutilisation of primary health facilities during childbirth and postnatal care services in the rural area of Tanzania. Does the quality of provided obstetric care matter?	International Centre for Reproductive Health, Ghent University, Ghent, Belgium	Finalisation of the research protocol
3	Male	Navrongo Health Research Centre, Navrongo, Ghana	Economist	Cost-effectiveness of clinical decision support system in improving prenatal and maternal care in Ghana.	Institute of Public Health, University of Heidelberg, Germany	Published results from the first study; On-going second study; plan to finish in December 2014
4	Female	Muhimbili University of Health and Allied Sciences, School of Public Health and Social Sciences, Dar Es Salaam, Tanzania	Economist	Improving prenatal and maternal care in Lindi and Mtwara regions in Tanzania: An economic evaluation of the proposed provider incentive scheme and the CDSS.	Institute of Public Health, University of Heidelberg, Germany	Paper under review on the first study; On-going second study; plan to finish in December 2014
5	Female	Institute of Public Health, University of Heidelberg, Germany	Social Scientist	Influences on and measurement of maternal and neonatal health provider motivation in low income settings.	Institute of Public Health, University of Heidelberg, Germany	Completed doctoral work in 2013
6	Female	Navrongo Health Research Centre, Navrongo, Ghana	Social Scientist	The effects of non-financial incentives on motivation and performance of reproductive health professionals in the Kassena-Nankana Districts of Northern Ghana.	Kwame Nkrumah University of Science and Technology, Ghana (this University is outside of the QUALMAT consortium)	On-going studies; first paper under development; plan to finish in 2015
7	Male	Centre de Recherche en Sant_e de Nouna, Nouna, Burkina Faso	Medicine	Performance-based incentives schemes for Health care providers in rural Nouna Health District: design, implementation, and effects on maternal and neonatal care results in Nouna Health District, Burkina Faso.	Institute of Public Health, University of Heidelberg, Germany	Paper under review on the first study; second paper under development; plan to finish in December 2014
8	Male	Centre de Recherche en Santé de Nouna, Nouna, Burkina Faso	Computer specialist	Monitoring health impact of the utilisation of CDSS for safe maternal and childhood health care management.	Division of Clinical Pharmacology, Department of Laboratory Medicine, Karolinska Institutet, Stockholm, Sweden	First paper published; plan to finish in 2015
9	Male	Navrongo Health Research Centre, Navrongo, Ghana	Mathematics	Effect of CDSS on workflow processes and maternal health outcomes in rural northern Ghana.	Department of Clinical Pharmacology and Pharmacoepidemiology, Medical Clinic, University Hospital of Heidelberg, Germany	First paper under development; on-going second study; plan to finish in 2015
10	Male	Muhimbili University of Health and Allied Sciences, School of Public Health and Social Sciences, Dar Es Salaam, Tanzania	Informatics	Training strategies and opportunities of a computerised CDSS for maternal and neonatal care in rural districts in Tanzania.	Department of Clinical Pharmacology and Pharmacoepidemiology, Medical Clinic, University Hospital of Heidelberg, Germany	First paper under review; on-going second study; plan to finish in December 2014

Each doctoral student had one main supervisor at the host institution, and two co-supervisors – one from the host institution and one from the consortium member institution in their home country. The second co-supervisor was, in most cases, the PI from one of the three sub-Saharan African countries involved. The PIs were responsible, not for the direct scientific tutoring but, for the overall planning of parallel studies within the respective country. The doctoral students were required to spend varying periods of time at their host university. This ranged from 3 to 4 months to 2 years depending on the internal regulations of the universities concerned. The rest of the time was spent on field work, generally in the home country conducting the interventions and assessments within the Work Packages.

This cross-sectional qualitative study was carried out with the doctoral students, supervisors and country PIs within the project. The questionnaire was initially developed to elicit information from the 10 doctoral students. The questionnaire was then adapted to elicit information from the eight people involved in their supervision, and further revised to gather information from the three country PIs to gather perspectives of the different stakeholders. The questionnaire can be found in the end of this paper where the differences between the versions of the questionnaires are illustrated (Table 2).

**Table 2:** Questionnaires for the supervisors and doctoral students

Questions for supervisors
**1. What were your expectations regarding the following**:
1.1 Your doctoral student(s)
1.2 The host university (the university, where the doctoral **student is registered**)
1.3 The sending institution or university
1.4 The support the doctoral student(s) have to receive whilst undertaking field work
1.5 The sponsor (EU)
**2. Have you experienced any difficulties – and if so, what kind?**
2.1 Difficulties linked to the scientific work of the doctoral students/to further education during the doctoral training of the students
2.2 Organisational difficulties
2.3 Financial difficulties
2.4 Difficulties related to your role as a supervisor
2.5 Others (please explain)
**3. Please estimate**
3.1 How long will your student(s) need to complete their doctorate successfully?
3.2 How many of the doctoral candidates within QUALMAT do you think will be successful? (%)
3.3 If you think that some will fail, what do you think the main reasons for this will be?
**4. Recommendations**
4.1 What would you do differently by the supervision of your next doctoral students?
4.2 What advice would you give to other supervisors in a similar situation (doctoral students supervision in the frame of an EU-funded project)?
4.3 What other recommendations would you make?
**5. Final points**
5.1 Are there any other issues you would like to raise?
**Questions for the doctoral students**
**1. What were your expectations regarding the following:**
1.1 Your supervisor
1.2 Your host university (the university, where you are registered)
1.3 Your sending institution or university
1.4 The support you received whilst undertaking field work
1.5 The sponsor (EU)
**2. Have you experienced any difficulties – and if so, what kind?**
2.1 Difficulties linked to your scientific work/your further education during the doctoral training
2.2 Organisational difficulties
2.3 Financial difficulties
2.4 Difficulties related to your role/status as a doctoral student
2.5 Others (please explain)
**3. Please estimate**
3.1 How long will you need to complete your doctorate successfully?
3.2 How many of the doctoral candidates within QUALMAT do you think will be successful? (%)
3.3 If you think that some will fail, what do you think the main reasons for this will be?
4. **Recommendations**
4.1 If you had the chance to repeat the process of starting your doctoral studies what would you do differently?
4.2 What advice would you give to other doctoral students in a similar situation (conducing their research in the frame of an EU-funded project)
4.3 What other recommendations would you make?
**5. Final points**
5.1 Are there any other issues you would like to raise?

The questionnaire focused on five main themes: 1) expectations and the extent to which these had been met from the perspective of students/supervisors/hosting university/sending institution or university/sponsor; 2) difficulties linked to the scientific work and to further education during the doctoral training, including organisational and financial difficulties, difficulties related to the role as a supervisor or student; 3) time estimated for completion of the doctoral work and possible reasons for delayed progress; 4) points the supervisors would pay attention to in the future and advice to other students or supervisors; and 5) other issues.

The sample included all 21 potential respondents (students, supervisors and country PIs) and the project manager distributed the questionnaires by email. The recruitment of the participants was done by an accompanying letter that explained the intention behind the questionnaires, encouraged honest criticism, stressed that the data would be anonymised before analysis, indicated that taking part would take up to 1 hour and the voluntary nature of participation was emphasised. All those that responded checked a box to give their informed consent, filled out the form electronically and returned it by email. Returned questionnaires were complete with no information missing. The questionnaires were collated, allocated a number and uploaded in NVivo9^®^ for content analysis. Coding was undertaken by working through the different questions and identifying themes directly from the responses (17). A student from the first and second authors’ faculty who was not involved in QUALMAT undertook the first round of coding to increase objectivity. NVivo9 made the process of coding transparent as the results can be readily exported and circulated (18). This facilitated an iterative process of joint interpretation and analysis by the project manager and supervisors.

Ethical clearance for the study (S-339/2013) was received from the Ethics Committee of the Medical Faculty of the University of Heidelberg, Germany.

### Limitations

The study while interviewing almost all relevant participants nonetheless reflected a small number of respondents. In addition, the analysis was undertaken by those involved in the project and those located in the North. This introduces the risk of bias, although steps were taken to mitigate them by using an independent person to carry out the preliminary coding.

## Results

Completed questionnaires were received from nine doctoral students, six doctoral supervisors, and three country PIs (85.7% response rate).

### Expectations of the doctoral students and supervisors

One of the overarching themes that emerged was that of diverging expectations. All of the doctoral students from SSA described high expectations about the technical input they would receive. These expectations spanned support with administrative issues such as registration at the host university, equipment (laptops, software, and books), and the amount of formal training (short courses) available to them. The term supervision was understood to mean active support with carrying out the literature review, accessing documents, preparing and submitting abstracts for conferences, and paper writing. There was a sense that precisely because the host university was in Europe there would be this high level of support. This led in some cases to disappointment and, in waiting for such support, time was wasted.

The main supervisors considered that the doctoral students from SSA lacked initiative at times. The responses indicated that supervisors understood this lack of initiative to be partly explained by what they considered to be doctoral students’ unrealistic expectations; their lack of familiarity with the context and prevailing administrative requirements and language problems. Several respondents mentioned that this might have been compounded by what they perceived as the students’ deference to hierarchy and authority. The staff at the European universities expected rather pushy, ambitious, highly organised individuals, characteristics that often distinguish students opting to continue to doctoral level studies in the European context. This cultural clash can be illustrated by an example: the students were not supported in all cases to get their visas for Europe. The European universities knew that they could only issue a letter of invitation and that each person had to get their own visa and the institutions in SSA assumed the host universities were responsible to get the visa. It took time for the students themselves to become active as there had been an expectation that this would be done for them. As a result some had to delay their travel. The relationship got off to a bad start as the European university contacts interpreted it as a sign of poor organisation or low interest on the part of both partner institutions and students, whilst the students’ initiation to their doctoral training was one of feeling unsupported and, even worse, unwelcome.

A further divergence emerged as all the main supervisors expected that doctoral students’ existing employment contracts would provide them with the necessary job security to commence their literature reviews and draw up their study protocols even whilst their formal university acceptance as doctoral candidates, and in-country ethical clearance for QUALMAT itself, remained outstanding. However, most of the students only started to work after their first visit to their European host university upon completion of the administrative requirements.

Some of the respondents suggested that given the divergence of backgrounds the project should have invested more energy in ensuring common understanding at the outset. It emerged that for many students the premise of doctoral level study being the production of original work had simply not been clear. The doctoral students recollected that early on, the sharing of proposals led to arguments about the ownership of ideas and about who could take advantage of some of the available quick-wins rather than discussions on how synergies could be optimised between the different studies. Moreover, some of the main supervisors were surprised that structured reporting of doctoral study progress did not form part of the required technical reporting to the EU.

It should also be noted that language issues caused problems. Although English was the national language or language of instruction for some of the doctoral students, the standard of their written English was not as high as expected by the host universities, something that was not picked up by the entrance requirements. This resulted in supervisors and other project collaborators investing additional effort in drafting and proofreading research plans, protocols, and papers beyond the expected technical review.

### Varying understanding of the rationale for nesting doctoral students in the project

The selection procedures applied and understanding of the purpose of the doctoral students in the project were further areas of considerable divergence. All the country PIs were very clear that they chose the candidates because of their general skills and the overall contribution they could make to QUALMAT and not specifically because they were well suited to further study. They preferred candidates from the institutions involved in the consortium and considered that the students increased knowledge and skills would be of wider benefit at their workplaces, as well as to increase the chances of their being retained after the project was completed. The supervisors at the host universities were not involved in the selection procedures, a fact that was later regretted. Amongst some there was disappointment about the level of academic performance and failure of candidates to meet the expected level of knowledge and abilities. There was concern that local politics and connections may have influenced the selection process. There were cases where the main supervisors were not very familiar with the home country of the doctoral student, a situation that all would reportedly seek to avoid in the future.

### Understandings of arrangements and responsibilities

The co-supervisors in the partner countries in SSA were generally responsible for many QUALMAT activities including the overall coordination for data collection within the doctoral studies. The former role was accorded far greater importance. With regards to the latter, there was an assumption that sufficient supervision would be provided by the host university. The urgency of the doctoral study timeframe was often not appreciated. Deadlines for PhD requirements were not initially even conveyed between the main and the co-supervisors because it was taken for granted. Furthermore, it was thought that the doctoral students would also receive funds from their host universities even though communication stressing that their costs had to be included in-country budgets were clearly made by the project manager. As a result, some doctoral students were not included in Work Package budgets or schedules, which was a further cause of difficulties. In some cases, the doctoral studies had to be adapted due to the lack of funding for even small-scale assistance with data collection.

Many of the doctoral students interpreted this situation as being a sign that the Country PIs were not supportive and they considered this a major barrier to timely progress with their studies. Conversely, the doctoral students who pushed harder for the inclusion of their own studies in Work Package budgets were sometimes perceived as selfish by the co-supervisors. Some of the doctoral students felt that they had less access than others to the data being generated by the project. The country PIs were rather critical of the extent to which these students focused upon their doctoral studies. They perceived these students to have prioritised their own agenda over the overall project interests.

In retrospect, all of the respondents felt that too little was done by the project to facilitate on-going communication between the main and co-supervisors. Furthermore, the public project website and intranet platform were only used as an exchange platform for literature and project products (study protocols, ethical clearance, results, reporting and publishing guidelines). There could have been further potential to use it for communication and exchange of information between the doctoral students on the progress of their studies. Many described how understanding of core concepts such as supervision, the expectations made of the doctoral students, their standing in the project, the importance of treating them equally as far as possible (given some differences in requirements between universities), and so on, varied and should have been clarified from the start. Some responses suggest an assumption that, simply because the doctoral studies were part of such a project, they would ‘somehow happen’. Given this, and because the EU reporting was focused upon the Work Packages, it was the project itself that held the focus of their attention. When communication about the doctoral studies was arranged, it was felt to be ad hoc and limited and could not solve acute difficulties – a case of too little too late. Whilst the students were in their home countries, the main supervisors in the North complained that they were left uncertain about student progress. This can be better understood in light of the perceptions of the country PIs revealed above. Cultural differences with regards to losing face, incurring the ire of the co-supervisors, and a fear of being thrown out of the programme appear to have limited the candidates’ willingness to alert their main supervisor about delays and difficulties in a forthright manner so that corrective action could be initiated in good time.

### Funding doctoral studies

The major concern mentioned by all respondents was that of funding. The budget of €36.000 set aside for each doctoral student was shown to be insufficient. This only covered a stipend per month for 3 years and did not extend to cover travel costs, field work, travel between the host university and home country, or travel to international conferences. It was intended that these additional expenses would be integrated into Work Package budgets; however, the funding was not ring-fenced for doctoral studies meaning that such possibilities dwindled over time. This caused frustration on the part of the main supervisors as they did not have the final say about their students’ budget. The co-supervisors reported having had a long period during which they were reluctant to include costs for the doctoral studies in Work Package budgets as they believed the European universities would find additional funds from ‘somewhere’. Only very late did they report having understood that this was not the case, a situation that endangered the activities of some of the doctoral students.

Economic difficulties forced all of the doctoral students to take on additional work during the timeframe of their studies. Many of them undertook such work within the project itself. However, this was not mentioned as being a source of delays. Rather it was appreciated as it generated a deeper understanding of the issues being researched. The project was also described as having a particular dynamic that nonetheless kept things moving forward and provided the doctoral students with deadlines. There were, however, also project delays. For example, the delay in implementing interventions needed to do before and after comparisons, and financing delays associated with the release of the tranches of project funds had repercussions for the doctoral studies. This was difficult for the doctoral students to deal with. It caused them to extend their time in their home countries, held up their progress for even minor issues such as travelling to the sites and engaging data collection support and caused them to become involved in other work, which took them completely away from their studies and delayed their overall progress.

Budgetary concerns were also described as having been a cause of resentment and jealousy between the doctoral students as some were seen to have benefited from attending international conferences or training whilst others did not. Even worse, the ill-feeling may have spilled over to staff at the facilities involved in the interventions. An example was cited of a doctoral student having spoken badly of the project and its lack of budget to the health workers involved in data collection. The level of the stipend itself was criticised by the recipients as no longer being in line with the cost of living in many European cities and therefore their stay at the host institution was shorter. The inadequate stipend was held responsible for the students not being able to socialise or integrate fully in their departments. This was something that many found difficult enough anyway given their frequent absences for field work. The students were all breadwinners for families and had expected that they would be able to send part of their stipend home. The Country PIs were rather dismissive of the student's complaints about budget with some citing the difficulties they themselves had suffered as young scientists in the same situation indicating that these challenges were somehow a ‘rite of passage’ that had to be endured.

## Discussion

The study provides a critical review of key issues for nesting and effective integration of doctoral students within major collaborative projects. The authors offer a checklist (Table 3) based on experiences from the QUALMAT project and results of this study. The checklist is designed for practical use from the very beginning of any programme involving students from SSA undertaking doctoral studies. The key challenges faced and areas where facilitating factors have been identified can be classified as follows: 1) applicant selection; 2) application and admission procedures; 3) supervisor–student responsibilities; 4) training curriculum; 5) cultural considerations; 6) budget and funding; and 7) dissemination. The section below discusses the main findings. Out of the study we provide a set of recommendations, linked to different levels and phases of the research project which are presented in a condensed form in Table 4. Reference to these recommendations would help to avoid, anticipate or prepare for the main challenges and in this way facilitate more efficient management of doctoral students in the future.

**Table 3:** Checklist of essential requirements for the nesting of doctoral studies in joint North–South projects

Before proposal development and submission	
1	Survey expectations of all project partners (responsibilities for administrative issues including travelling and visa issues, access to information/libraries, technical infrastructure, drafting and reviewing of study protocols, reports and papers, feedback from tutors, prioritisation and support of research project with respect to competing tasks)
2	Agree on process of identification and selection of suitable doctoral candidates
3	Specify doctoral requirements of host university (minimum duration, required presence at the university, achievements relevant for grading, role of student and tutor)
4	Clarify financial requirements and budget required for doctoral student salary training and research (e.g. covering living expenses at home and longer stays at host university, travelling, and communication)
5	Establish state-of-the-art teleconferences and define incentives/rewards to establish frequent and effective North–South information exchange
6	Agree on principles of information exchange and standards of communication
7	Agree on standards of doctoral project management (meetings, deadlines, and milestones)
During proposal development and submission	
1	Feasible technical proposal for doctoral studies
2	Check training status and organise courses to fill relevant gaps (project management, scientific writing, computer and language skills)
3	To consider differences in administrative and financial requirements of the hosting university for final budget allocation for the doctoral students
4	Make sure that co-PIs located in the country, in which a study is planned and conducted, critically review any protocol, commit to the plans in writing, and take joint responsibility for the conduct of the study.
5	To develop a detailed plan for each of the doctoral studies (including required trainings, study activities, writing activities, travel to host institutions, etc.)
6	Call for full application of students with degrees, CVs and letter of recommendation. Doctoral students must present former research work
7	Submit the study protocol to the local ethical authority at the study site and to the ethical commission of the host institution
During conduct of the project	
1	Create a platform where all doctoral students discuss their work regularly among each other (platform managed by doctoral students themselves)
2	Define possible additional duties of doctoral students within the project, within the home and hosting institution
3	Make a clear mapping of all doctoral studies within the project and how they are linked
4	Plan carefully the visits to the institutions for field work, so that they are not overwhelmed with the doctoral students
5	Assure regular submission of mid-term reports from the students on their research progress, presentation activates, and status of the publications
Three to four months before the end of the project	
1	Plan for the completion of all doctoral studies at least couple of months before the end of the main project
2	Check again all administrative requirements regarding thesis submission, exam, required publications
3	Secure financing until completion of the doctoral studies

**Table 4:** Set of recommendations for nesting and management of doctoral students within major collaborative projects

Focus	Main recommendation (to avoid key challenges and promote facilitating factors)
Applicants selection, application procedure	Give priority to the preferences of the partner countries, where the research will take place.
	Launch a competitive call for the selection of doctoral students with a rigorous screening modality for proving the capacity and skills of the candidates.
	Encourage the selection of doctoral students with different backgrounds and to facilitate regular exchange of information and expertise between them later.
Students admission, registration, and employment	Encourage the further employment of the students within their home institution despite their registration as doctoral students at the host university.
	Use the ‘sandwich’ programme mode for efficient use of resources, meaning main employment at the home institution with several visits for a couple of months to the host university.
	Have formal induction procedure (clear instructions, specify responsibilities and rights of the students) to the host university immediately after the registration.
	Encourage the registration of the students at African partner university, which would support further institutional capacity development. There is a need for greater advocacy regarding the prestige of science and degrees that are generated and gained in Africa.
Student management, mentor–mentee responsibilities and training curriculum	Consider management of the student's expectations alongside clarification of their responsibilities and to encourage their proactive role.
	Consider development and approval of structured research plan from the very beginning in order to facilitate their integration in the Work Packages plans.
	Set up a monitoring (milestones) and evaluation system for all doctoral students with reporting on the progress of doctoral studies and the steps taken towards such integration.
	Provide an extensive coaching of the doctoral students through a bi-national team of a main and co-supervisor (at the place of field work) with clear responsibilities for all sites. To improve the doctoral students’ knowledge through utilisation of the experience of local experts.
	Locate, if possible, the doctoral studies predominately in the pre-intervention project phase, which would give time advantage and avoid the challenges that pre–post intervention studies can present.
Cultural considerations	Treat all doctoral students equally as far as the differences in requirements between universities allows.
Project management, budget and funding	Ensure strong coordination and strict reporting systems, clear communication with all stakeholders regarding the project's progress, and annual face-to-face consortium meetings and/or regular phone conference discussions.
	Clear assign funding to the doctoral students from the time they started developing their proposal until their actual submission.
Dissemination	Ensure the participation of the students to scientific conferences.
	Have written publication guideline as a standard practice, which is of particular importance to guide doctoral candidates.

The careful selection of applicants is essential for successful doctoral studies. Candidates from the partner countries were preferred so that a sub-Saharan perspective would help shape the project's emerging research agenda (4), although simply expecting this to happen was found not to be enough. A meaningful involvement would have required more active facilitation and encouragement. Young and mid-level candidates were sought as the current population of African researchers is ageing (19). However, the cultural issues surrounding the requirement of younger people to show respect to age and authority was raised indirectly as an impediment to the doctoral students on several occasions, and has also been highlighted by others. Maina-Ahlberg and colleagues (20) raised the concern of whether merit was the only criterion used in such selection processes (20). QUALMAT did not use the website to launch a competitive call for the selection of doctoral students although, with a more rigorous screening modality, it may have held such potential.

The importance of inter-disciplinary exchange has long been recognised in public health and is even more crucial when researchers are in low supply overall (21). This was confirmed within this project, where we have students with a wide variety of backgrounds. The advantages of encouraging such exchange at doctoral level are to be particularly noted. There were also discipline-specific issues that arose: specifically, given the focus upon health worker motivation and incentives which require an in-depth understanding of the culture and context, the doctoral studies of the social scientists focused on the local context offering limited potential for cross-country comparison.

Whilst it became clear that uniform selection procedures would have been beneficial and the supervisors at the host university should have been more closely involved, leaving the selection to the country PIs did have some advantages. These included that the candidates came from a variety of backgrounds including medicine, nursing, social sciences, economics, mathematics and informatics, which facilitated a multidisciplinary approach. They were also all known to be reliable and most of them were identified early on so they could take part in all initial discussions and planning during the international/national project kick-off meetings. Moreover, the candidates were anchored at the African partner institutions from the outset making it more likely that they would remain there upon completion. Despite the high risk of researchers from SSA being drawn to relocate to countries where their efforts are better rewarded (19), the young researchers have all remained in-country which is an achievement in itself.

Despite the steps taken to find good candidates, delays to ‘get things moving’ at the beginning were still experienced. With hindsight it was agreed that a formal induction procedure would have been useful (10). There seemed to be an assumption that there is a universal standard for doctorate administration and admission and that the European standard is the norm. Greater sensitivity about this with focused efforts to assist foreigners would have been helpful. Many of these issues could be addressed by providing clear instructions on the steps to be taken from the point of registration until the completion of the doctorate. By also specifying responsibilities and outlining what should have been completed before a student could move forward to the next stage would have managed expectations better. The early development of structured plans for doctoral studies and proper integration in the Work Package plans would have reduced later misunderstanding. The EU could consider encouraging reporting on the progress of doctoral studies and the steps taken towards such integration. Ways of conveying the importance of starting such activities and communication and reporting mechanisms should have been put in place from the start.

Regarding supervision, some of the European institutions involved had more experience in receiving and supervising students from overseas. The importance of sharing such experiences was underestimated at the outset but later addressed in part due to the study findings. The concept of establishing a team of main and co-supervisors to provide tutoring to the doctoral students has further potential and should be considered. The building of bi-national supervisory teams was undertaken to make sure that the requirements of both the host and home university were observed and that the mentoring could take context specific challenges into consideration. In-depth knowledge about conditions in SAA study such as access to journals and internet bandwidth to facilitate searches – easily available and at no cost to students located in European universities yet significantly restricted or non-existent for researchers in SSA – and developing ways of redressing this rather than describing students from SSA as needy could also improve supervisor–student interactions. Nesting students did provide a modality for overcoming the poor supervisor to student ratio found at African research centres (10). Nonetheless, the extensive coaching the doctoral students required was underestimated and more main supervisor time should have been included in the planning.

The European universities and the university level institutions involved in SSA all offer an array of basic and advanced short courses in the area of public health as part of their core mandate. The doctoral students were found to be in particular need of training on issues like how to conduct a literature review, use of electronic databases, how to undertake qualitative research, and how to write a research protocol or scientific paper. Paper writing management plans would have reduced unclear expectations for all concerned. Due to budgetary concerns the extent to which the doctoral students could take advantage of existing courses was limited. There were, however, some good examples with a joint training organised by two of the Work Packages on how to use NVivo9 to facilitate the analysis of qualitative data. Care was taken to time this course so that pre-test data generated by tools developed by the doctoral students themselves was available for the training.

The proactive role of doctoral students should be encouraged under all circumstances. The experiences also underline the importance of treating all doctoral students equally as far as differences in requirements between the universities involved allow. Greater attention should have been given to these aspects from the start. In addition, the findings show that the consortium partners involved from SSA have to define the role of their doctorate students more fully and to move beyond the mere approval and monitoring of project activities at the respective sites. This would assure closer supervision and allow doctorate students to learn from the experience of local experts.

Although some of the doctoral students were admitted early on in the project this did not automatically translate into a quick start to their studies. For some Work Packages, it took quite some efforts to get the doctoral studies up and running and in the end some studies could only be carried out pre-intervention as the timelines overall had slipped. It would have been advisable for the doctoral students to have located their studies in the preliminary pre-intervention project phase. This would have given them a time advantage and further ensured that they avoided the challenges that pre–post intervention studies can present.

Robust overall project management is essential for doctoral studies undertaken in the framework of collaborative projects. Components that may be considered particularly important include: strong coordination and strict reporting systems to ensure that all the Work Packages kept broadly to time, clear communication with all stakeholders regarding the project's progress, and annual face-to-face consortium meetings/regular phone conference discussions.

Greater clarification regarding the division of the costs for the doctoral students between their host and home institutions would have been helpful at the outset. Collaborative projects always need to carry out a careful budgeting of the planned interventions and on-going parallel field studies. It is crucial that the full dimension of the costs of doctoral studies, including scholarships, travelling, conference presentations, paper submission, and training activities, are taken into account. In this project, despite the modest demands of the doctoral candidates, the costs of enabling all of them to travel to meetings/conferences were underestimated in the country budgets. A clear assignment of funding to the doctoral students from the time they started developing their proposal until their actual submission would have avoided later confusion about entitlements to the stipend from home institutions.

The interruptions to doctoral research that occurred due to delays in the release of project funds from the EU should also not be underestimated. The administrative formalities and the procedure for financial reporting are understandably strict in collaborative research projects. For the EU to release the next tranche of funds, the technical and financial reports of all six universities involved needed to be accepted. If just one institution has a problem this has implications for all the partners. There were several such delays and these were difficult for the institutions in SSA to manage. In particular the smaller of these research institutions with limited internal flexible funding the scientific work, including the work of the doctoral students, was directly affected. This put some of the doctoral students at risk of not being able to complete within the planned 3-year period.

Annual project meetings could be considered as a key forum for collaborators to receive feedback from the Scientific Advisory Board and relevant stakeholders. In our case, meetings of the doctoral students arranged around the annual meetings within the project proved to be an opportunity to facilitate peer exchange and support. Other researchers also confirmed this, especially with regards to fieldwork (22), opportunities to seek student support (10) and post-doctorate career planning. Another facilitating factor for the progress of the doctoral candidates is their regular participation in different scientific forums, conferences, and training. Gaining access to courses is known to be a major deficit for students from SSA (23) but also at European universities, because of the high cost. In addition, the use of low cost technologies such as Skype, video and phone conferences can facilitate regular communication between the project collaborators despite some technical problems.

The existence of a written publication guideline is standard practice within such collaborative projects, but may be considered of particular importance to guide doctoral candidates. The role of on-site support from individual members of the Scientific Advisory Board complemented the Country PIs and Work Package leaders in providing good internal peer review of papers from doctoral candidates before journal submission, and is highly recommended.

Whilst most of the doctoral candidates were from the partner countries in SSA, all except one of them were enrolled at European universities. This clearly runs the risk of only building health research capacity at the level of the individuals involved. Although steps were taken to make sure the candidates were linked to research institutions in the South, it would have been more ambitious to have had more of the candidates enrolled at SSA institutions particularly Muhimbili University of Health and Allied Sciences which as a University, could have taken on this role. This would have increased the development of institutional capacity in the South. Unfortunately, it is also possible that a degree of prestige remains attached to gaining a doctorate at a European university. There is a need for greater advocacy regarding the prestige of science and degrees that are generated and gained in Africa. There are several published findings on good practice and international partnerships in research and training, where the lead is taken entirely by organisations in SSA and there are visible research outputs (4, 5, 24).

Apart from the big collaborative projects as a platform for doctoral student's recruitment and management, special doctoral programmes or partnerships have particular roles in building research capacity. The challenges surrounding their establishment and sustainability have been well recognised by several researchers. These include lack of political will and research leadership (23), growing numbers of students and low number of academic staff (10), absence of legislation, poor institutional level capacity, limited experience in handling research funds (7), lack of investment and infrastructure (11, 25).

## Conclusions

This study presents the challenges of nesting doctoral students in major collaborative projects. It also provides insights on how the QUALMAT project found ways to overcome these challenges, recommendations, and a checklist to guide other institutions involved in projects where students from SSA are engaged in doctoral level studies. The checklist in particular has been designed for use from the very beginning of project conceptualisation and planning.

Major collaborative projects like QUALMAT provide important scope for the fostering of close partnerships among universities, health research centres, National Health Authorities, and health facilities. This can facilitate the strengthening of a research agenda that is led by, and builds local research capacity in SSA, whilst also strengthening North–South partnerships. The QUALMAT experience shows that such projects afford doctoral students unique benefits by engaging them in an international working environment whilst also bringing them in touch with senior policy advisors in their own countries and the wider region. Despite the reported constraints, overall the project consortium has been highly successful in reaching all important targets for the planned studies and scientific publications. The substantial contribution that the doctoral students have made to this success is emphasised. This is an important finding for all those interested in improving the planning and efficiency of managing doctoral students in major collaborative projects.

Through their doctoral studies at the host university and through the project itself, it is hoped that the doctoral students themselves will become ‘ambassadors’ for improving doctoral level opportunities in SSA and the recognition thereof. Whilst such efforts do not replace the importance of capacity development at the institutional level – comprising political vision as well as long-term systematic support and investment – they can usefully complement it.

## References

[1] ECA, AU, AfDB, UNDP. MDG Report (2012). http://www.uneca.org/sites/default/files/publications/mdgreport2012_eng.pdf.

[2] Kwesigabo G, Mwangu MA, Kakoko DC, Killewo J (2012). Health challenges in Tanzania: context for educating health professionals. J Public Health Policy.

[3] Fonn S (2011). Linking public health training and health systems development in sub-Saharan Africa: opportunities for improvement and collaboration. J Public Health Policy.

[4] Zumla A, Huggett J, Dheda K, Green C, Kapata N, Mwaba P (2010). Trials and tribulations of an African-led research and capacity development programme: the case of EDCTP investments. Trop Med and Int Health.

[5] Ezeh AC, Izugbara CO, Kabiru CW, Fonn S, Kahn K, Manderson L (2010). Building capacity for public and population health research in Africa: the consortium for advanced research training in Africa (CARTA) Model. Glob Health Action.

[6] Mgone C, Volmink J, Coles D, Makanga M, Jaffar S, Sewankambo N (2010). Linking research and development to strengthen health systems in Africa. Trop Med Int Health.

[7] Kellerman R, Kilpstein-Grobusch K, Weiner R, Wayling S, Fonn S (2012). Investing in African research training institutions creates sustainable capacity for Africa: the case of the University of the Witwatersrand School of Public Health master's programme in epidemiology and biostatistics. Health Res Policy Syst.

[8] Macfarlane SB, Kaaya EE (2012). Universities in transition to improve population health: a Tanzanian case study. J Public Health Policy.

[9] Bates I, Akoto AYO, Ansong D, Karikari P, Bedu Addo G, Critchley J (2006). Evaluating health research capacity building: an evidence-based tool. PLoS Med.

[10] Bates I, Phillips R, Martin-Peprah R, Kibiki G, Gaye O, Phiri K (2011). Assessing and strengthening African universities’ capacity for doctoral programmes. PLoS Med.

[11] Lenters LM, Cole DC, Godoy-Ruiz P (2014). Networking among young global health researchers through an intensive training approach: a mixed methods exploratory study. Health Res Policy Syst.

[12] Mayhew SH, Doherty J, Pitayarangsarit S (2008). Developing health systems research capacities through north-south partnership: an evaluation of collaboration with South Africa and Thailand. Health Res Policy Syst.

[13] Thornicroft G, Cooper S, Van Bortelel T, Kakuma R, Lund C (2012). Capacity building in global mental health research. Harvard Rev Psychiatry.

[14] DFG German Research Foundation http://www.dfg.de.

[15] Prytherch H, Kagoné M, Aninanya GA, Williams JE, Kakoko DC, Leshabari MT (2013). Motivation and incentives of rural maternal and neonatal health care providers: a comparison of qualitative findings from Burkina Faso, Ghana and Tanzania. BMC Health Serv Res.

[16] Blank A, Prytherch H, Kaltschmidt J, Krings A, Sukums F, Mensah N (2013). “Quality of prenatal and maternal care: bridging the know–do gap” (QUALMAT study): an electronic clinical decision support system for rural Sub-Saharan Africa. BMC Med Inform Decis Mak.

[17] Hsieh HF, Shannon SE (2005). Three approaches to qualitative content analysis. Qual Health Res.

[18] Welsh E (2002). Dealing with data: using NVivo in the qualitative data analysis process. FQS.

[19] Whitworth JA, Kokwaro G, Kinyanjui S, Snewin VA, Tanner M, Walport M (2008). Strengthening capacity for health research in Africa. Lancet.

[20] Maina-Ahlberg B, Nordberg E, Tomson G (1997). North-South health research collaboration: challenges in institutional interaction. Soc Sci Med.

[21] Leshabari S, Lubbock LA, Jaijage H, Kalala W, Koehler G, Massawe S (2012). First steps towards interprofessional health practice in Tanzania: an educational experiment in rural Bagamoyo district. J Public Health Policy.

[22] Fyfe M (2012). Education projects: an opportunity for student fieldwork in global health academic programs. J Public Health Policy.

[23] Lansang MA, Dennis R (2004). Building capacity in health research in the developing world. Bull World Health Organ.

[24] de-Graft Aikins A, Arhinful DK, Pitchforth E, Ogedegbe G, Allotey P, Agyemang C (2012). Establishing and sustaining research partnerships in Africa: a case study of the UK–Africa Academic Partnership on Chronic Disease. Global Health.

[25] Lentera LM, Cole DC, Ruiz PG (2014). Networking among young global health researchers through an intensive training approach: a mixed methods exploratory study. Health Res Policy Syst.

